# Enhanced T cell receptor specificity through framework engineering

**DOI:** 10.3389/fimmu.2024.1345368

**Published:** 2024-03-12

**Authors:** Aaron M. Rosenberg, Cory M. Ayres, Angélica V. Medina-Cucurella, Timothy A. Whitehead, Brian M. Baker

**Affiliations:** ^1^ Department of Chemistry and Biochemistry and the Harper Cancer Research Institute, University of Notre Dame, Notre Dame, IN, United States; ^2^ Department of Chemical and Biological Engineering, University of Colorado Boulder, Boulder, CO, United States

**Keywords:** T cell receptor, specificity, protein engineering, molecular dynamics, framework regions

## Abstract

Development of T cell receptors (TCRs) as immunotherapeutics is hindered by inherent TCR cross-reactivity. Engineering more specific TCRs has proven challenging, as unlike antibodies, improving TCR affinity does not usually improve specificity. Although various protein design approaches have been explored to surmount this, mutations in TCR binding interfaces risk broadening specificity or introducing new reactivities. Here we explored if TCR specificity could alternatively be tuned through framework mutations distant from the interface. Studying the 868 TCR specific for the HIV SL9 epitope presented by HLA-A2, we used deep mutational scanning to identify a framework mutation above the mobile CDR3β loop. This glycine to proline mutation had no discernable impact on binding affinity or functional avidity towards the SL9 epitope but weakened recognition of SL9 escape variants and led to fewer responses in a SL9-derived positional scanning library. In contrast, an interfacial mutation near the tip of CDR3α that also did not impact affinity or functional avidity towards SL9 weakened specificity. Simulations indicated that the specificity-enhancing mutation functions by reducing the range of loop motions, limiting the ability of the TCR to adjust to different ligands. Although our results are likely to be TCR dependent, using framework engineering to control TCR loop motions may be a viable strategy for improving the specificity of TCR-based immunotherapies.

## 1 Introduction

T cell receptors (TCRs) orchestrate cellular immunity by recognizing short peptide antigens bound and presented by proteins of the major histocompatibility complex (MHC). TCRs on cytotoxic T cells recognize peptides presented in the context of class I MHC proteins and, in addition to other T cell effector functions, coordinate killing of the presenting cell. Accordingly, there has been substantial interest in developing TCR-based immunotherapies for cancer and infectious disease. Therapeutic approaches under development include cell therapies and soluble, bispecific constructs that link TCR recognition domains to antibodies (1).

Despite considerable progress in immunotherapy, TCR cross-reactivity remains a barrier to further advances. TCRs do not possess the high specificity of mature antibodies, with each TCR estimated to recognize and initiate responses against a million or more different peptide/MHC targets on average (2–4). Biologically, this high level of cross-reactivity emerges from the finite size of an individual TCR repertoire compared to the vastly larger number of possible peptide antigens. Although necessary to ensure our immune systems can effectively respond to evolving and emerging threats, cross-reactivity also introduces the risk of off-target recognition in TCR-based therapeutics.

A variety of protein engineering approaches have been explored to enhance TCR specificity. Early efforts attempted to mimic antibody maturation through phage or yeast display (5–8). However, because TCRs recognize a composite ligand comprised of both the peptide and the MHC protein, unlike what occurs with antibodies, strengthening TCR affinity towards a single peptide target does not always improve specificity: even with peptide-focused mutations, enhancing the physicochemical “fit” towards one peptide is likely to also improve the fit with others, bringing previously unrecognized peptides into an affinity window strong enough to elicit T cell responses (9). Indeed, in an early clinical trial, a TCR that was affinity enhanced towards a melanoma-associated peptide triggered off-target immune responses that lead to patient deaths (10, 11). Other TCR engineering approaches have directly focused on specificity. For example, structure-guided design that combines positive, peptide-focused mutations with negative, MHC-focused mutations has been explored as a way to “focus” TCRs on a single target peptide (12). Yeast display has been used to refocus a TCR from one peptide to another, yielding high specificity towards the new target (13–15).

While TCR engineering approaches have relied mostly on TCR mutations in the traditional, structurally defined TCR-peptide/MHC interface, introducing mutations outside of the interface is an alternative means to potentially alter or improve TCR specificity. This possibility is highlighted by the recent use of deep mutational scanning to study TCR binding, which demonstrated that mutations in framework regions distant from the interface could alter affinity through conformational, dynamic, or other long range effects (16). Such mutations may be advantageous in that they avoid directly altering the chemical makeup of the binding site, thus reducing the risk of introducing new reactivities. Indeed, although the targets of antibodies and TCRs are very different, framework sites have long been known to influence antibody loop conformational and dynamic properties (17–19), and mutations in framework regions above CDR loops have been associated with the generation of more potent and specific antibodies (20–23).

Here we explored the possibility of using framework mutations to improve TCR specificity. Studying the well-known Z11 variant of the 868 TCR specific for the HIV SL9 epitope presented by HLA-A*02:01 (HLA-A2), we identified candidate mutations in and away from the interface using deep mutational scanning. Probing two mutations in detail showed the framework mutation, a glycine-to-proline mutation above the CDR3β loop, led to improved specificity, as demonstrated by analysis of variants of the SL9 peptide as well as an SL9-based positional scanning library. Notably, the improvement in specificity was achieved without discernably impacting TCR binding affinity for the SL9 ligand or the TCR’s functional avidity in cellular assays. Molecular dynamics simulations suggested this mutation acted by reducing the fluctuations of the CDR3β loop, hindering its ability to structurally adjust to different peptides. Although limited at this point to a single TCR, the results of our study provide impetus for exploring non-interfacial, framework mutations as a means to enhance TCR specificity without altering binding affinity or introducing new reactivities through interface modification.

## 2 Results

### 2.1 Deep mutational scanning of a yeast displayed single chain TCR variant

To identify interfacial and framework mutations of interest, we performed deep mutational scanning of the Z11 variant of the 868 TCR. 868-Z11 incorporates multiple mutations which enhance the affinity of the 868 TCR towards SL9/HLA-A2 by 35-fold and stabilize a single chain variant, both of which facilitate deep mutational scanning of yeast displayed TCRs (24). Yeast display libraries, one covering the α chain and one covering the β chain, were generated via nicking mutagenesis. Primers encoding NNK codons were used to generate libraries of single mutations within the 868-Z11 scTCR in the pETConNK vector (25). The libraries encoded 778 and 839 mutations in the α and β variable domains, respectively. Mutations were made in all six CDR loops, covering the entire loops as well as amino acids in the pre- and post-loop framework regions. Libraries were transduced into *S. cerevisiae* for display and selection of mutants. A separate transformation was performed with the unmutated (referred to as WT) 868-Z11 single chain TCR.

**Figure 1:**
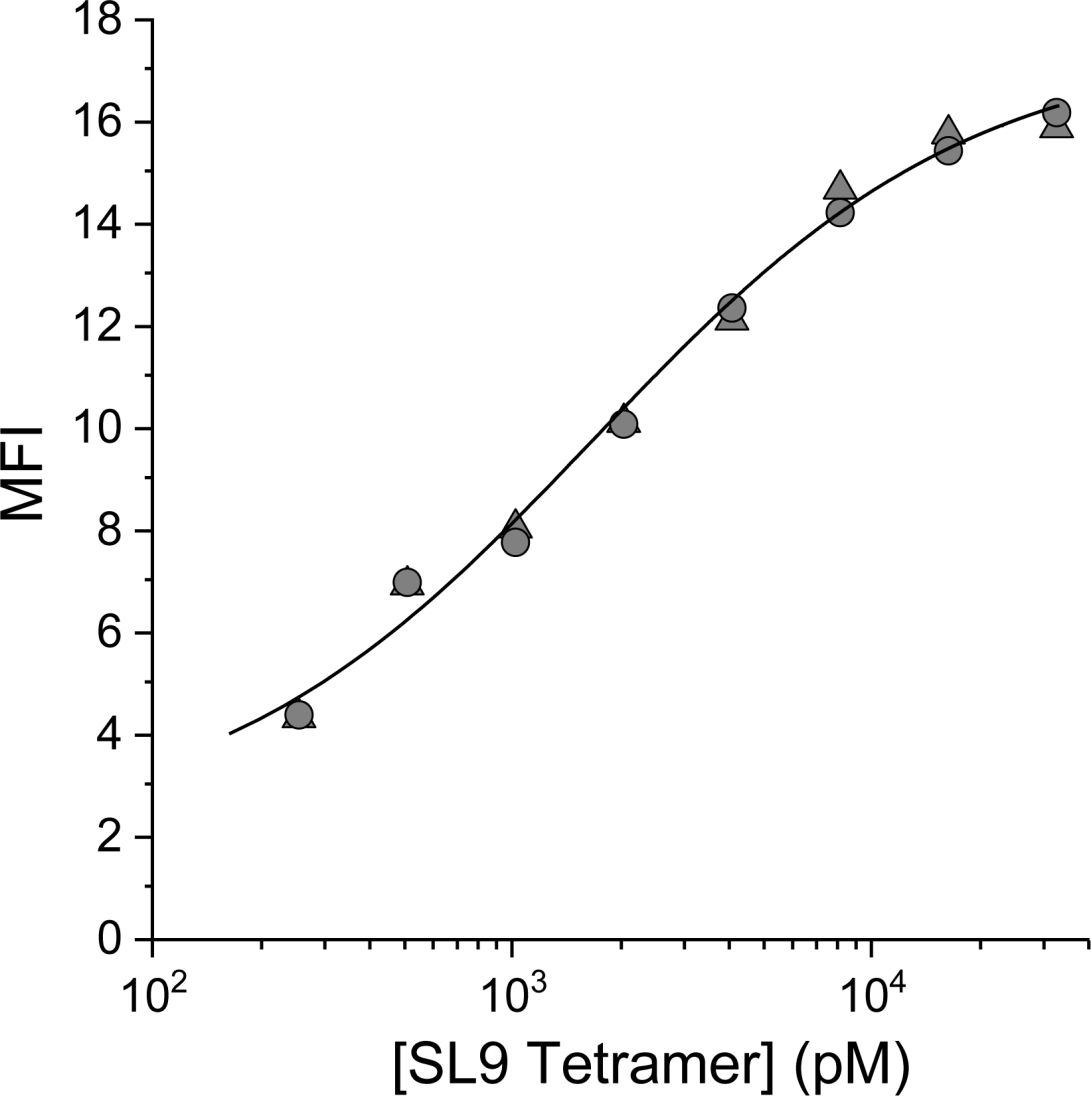
Yeast titration confirms the high affinity of the 868-Z11 TCR variant for SL9/HLA-A2. The data shows the MFI of yeast expressing the 868-Z11 scTCR stained at 4°C with PE-conjugated SL9/HLA-A2 pMHC tetramer at concentrations from 256 pM to 32.8 nM, with the titration performed in duplicate. The resulting curve was fit to a 1:1 binding isotherm, yielding a *K*_D,app_ of 1.7 ± 0.6 nM.

To determine the optimal concentration of SL9/HLA-A2 tetramer for staining the 868-Z11 yeast display library, we determined the apparent dissociation constant (*K*_D,app_) of the WT 868-Z11 scTCR-transduced yeast. WT 868-Z11 expressing yeast were stained with PE-conjugated SL9/HLA-A2 tetramer at concentrations ranging from 256 pM to 32.8 nM and subsequently analyzed via flow cytometry. The mean fluorescent intensity (MFI) was then fit as a function of tetramer concentration to a single site binding isotherm. This yielded a *K*_D,app_ of 1.7 nM (**Figure 1**), closely matching the previously determined value of 4 nM, similarly determined through cell staining (24). Although these values are likely influenced by the use of multi-valent, tetrameric reagents, prior work has shown good agreement between yeast titrations and solution measurements (24, 26, 27), possibly due to limited engagement of multiple ligands as well as a tradeoff between avidity effects and staining conducted at a reduced temperature, as performed here.

**Figure 2:**
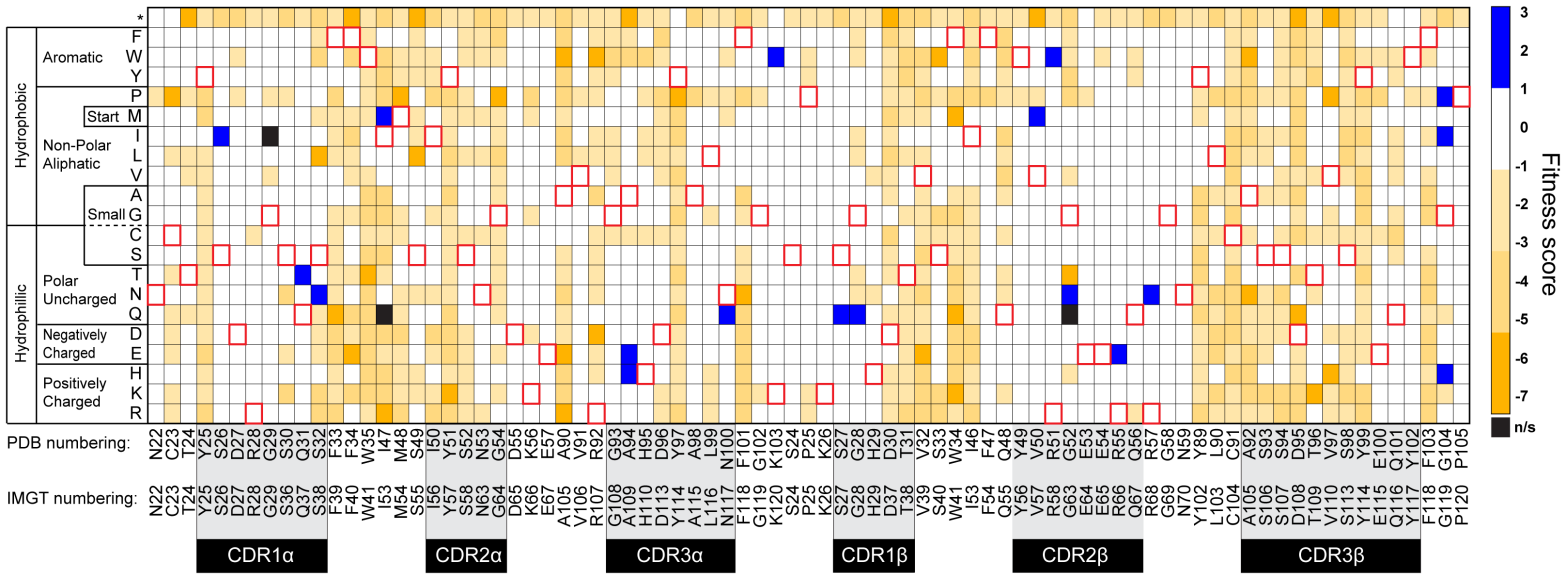
Heatmap of 868-Z11 deep mutational scanning data. Blue cells indicate a positive fitness mutation, orange cells indicate a negative fitness mutation, white cells indicate fitness mutations that are approximately equivalent to WT 868-Z11, and black cells indicate mutations that were not significantly represented in the sequencing data (indicated as n/s). Red outlines indicate WT amino acids. CDR loops are indicated by the shaded regions below the WT 868-Z11 sequence. Framework sites are those outside of the shaded regions. Amino acid numbering is per that found in the PDB file as well as according to IMGT standards, as indicated.

Based on the apparent *K*_D_, the 868-Z11 deep mutational scanning library was stained with 1 nM SL9/HLA-A2 tetramer as well as with an anti-c-Myc antibody. The anti-c-Myc antibody screens for full-length scTCR, preventing selection of partial constructs resulting from nonsense mutations generated from the NNK codons used in library production. After staining, the top 25% of the tetramer/antibody double positive population was sorted and grown. A reference population was also collected to determine the enrichment of mutants after selection (**Supplementary Figure S1**). The selected and reference populations were then deep sequenced using paired-end sequencing (**Supplementary Table S1**). The deep sequencing data was then used to determine enrichment and fitness values using the protein analysis and classifier toolkit (PACT) (28). The results are shown as a heatmap of fitness values in **Figure 2**. As expected, most mutations were deleterious, with only a small number of positive fitness mutations interspersed throughout the CDRs and framework region of the TCR.

### 2.2 Selection and evaluation of interfacial and framework mutations

**Figure 3:**
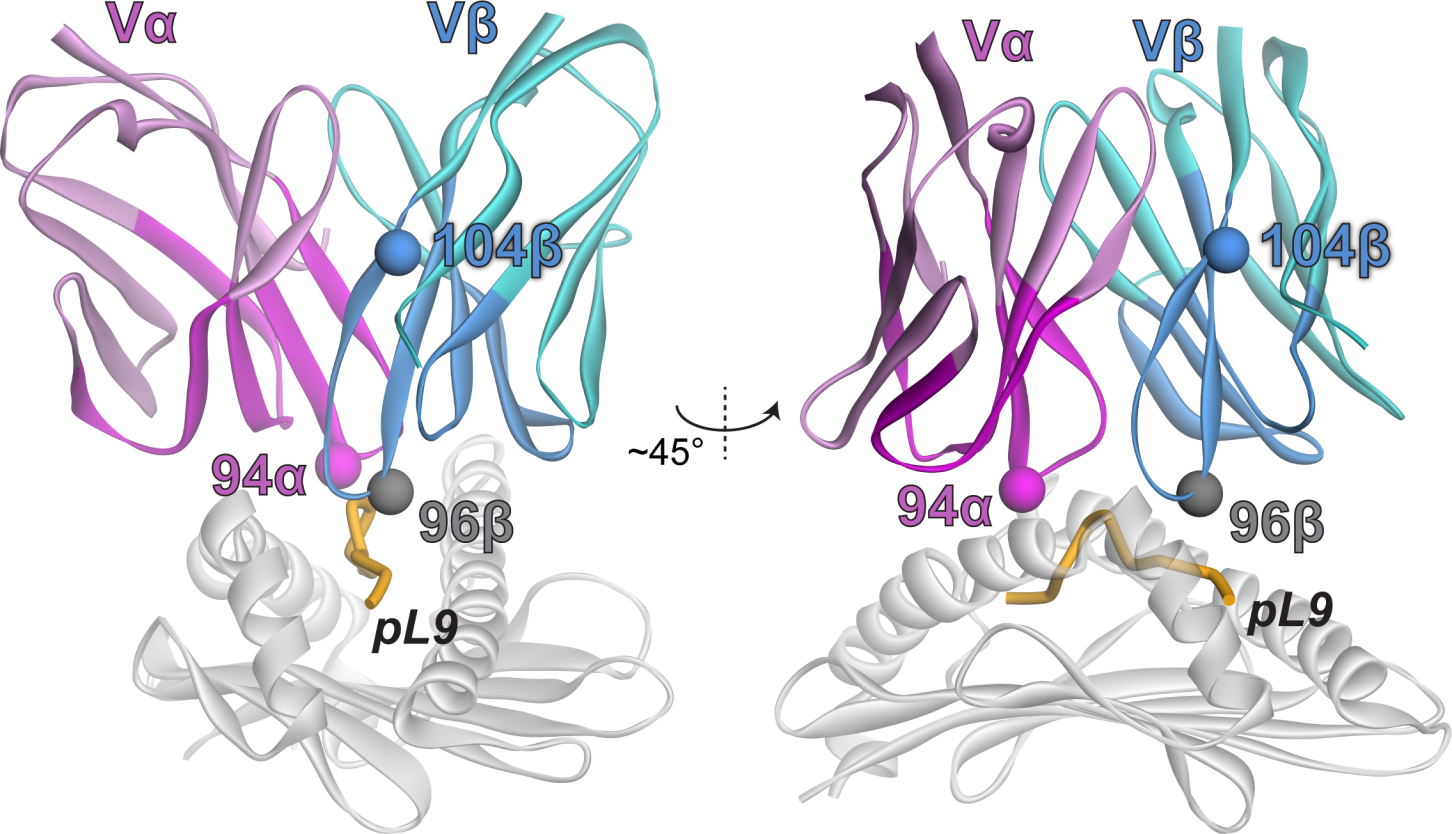
Locations of the selected mutations in the 868-Z11 TCR in the TCR-SL9/HLA-A2 complex. The α chain is magenta; the β chain is teal. Darker colors indicate residues that were included in the deep mutational scanning.

Mutations with positive fitness values were present in the CDR1α, CDR3α, CDR1β, and CDR2β loops as well as the framework regions flanking the CDR2/CDR3 loops. After examining each mutation in the context of the structure of the 868 TCR bound to SL9/HLA-A2 (29), we selected two mutations to evaluate further. The first was alanine 94 to histidine near the apex of CDR3α (referred to as A94αH using the numbering in the PDB file for the 868-Z11 TCR; this is equivalent to A109αH using IMGT numbering as indicated in **Figure 2**). The second was glycine 104 to proline in the β framework region following the C-terminal end of CDR3β (referred to as G104βP; equivalent to G119βP using IMGT numbering) (**Figure 3**). The interfacial A94αH mutation was selected to focus on CDR3 amino acids which contact the ligand and preferentially interact with the peptide vs. the HLA-A2 protein. The G104βP mutation was chosen as it represents a distal framework mutation that makes no short- or long-range contacts with SL9/HLA-A2, with the hypothesis that the glycine-to-proline mutation could alter binding indirectly by influencing the conformational properties of the CDR3β loop instead of directly altering peptide or HLA-A2 contacts.

**Figure 4:**
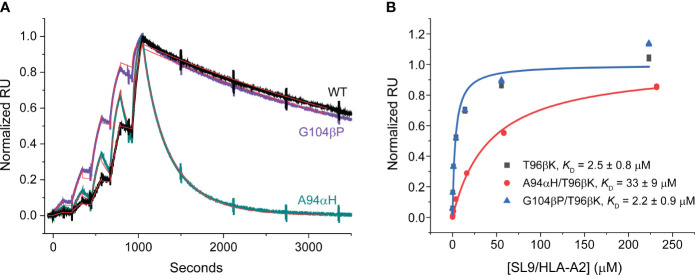
Binding data for the 868-Z11 TCR and mutants. **(A)** Single cycle kinetic titrations of SL9/HLA-A2 binding 868-Z11 WT (black), A94αH (teal), and G104βP (purple). Fits are shown as red lines. On rates (*k*_on_) were determined as 1.96×10^5^ M^-1^ *s* ^-1^ (WT), 7.92×10^5^ M^-1^ *s* ^-1^ (A94αH), and 9.04×10^5^ M^-1^ *s* ^-1^ (G104βP). Off rates (*k*_off_) were also determined as 2.16 x 10^-4^ *s* ^-1^ (WT), 4.15 x 10^-3^ *s* ^-1^ (A94αH), and 2.30 x 10^-4^ *s* ^-1^ (G104βP). From the on and off rates, the *K*_D_ values were determined as 1.1 nM (WT), 5.2 nM (A94αH), and 0.3 nM (G104βP). Large spikes associated with injections and pump refills were edited out for clarity. Data are reflective of two separate titration series. **(B)** Steady state titrations of SL9/HLA-A2 binding 868-Z11 T96βK (black), A94αH/T96βK (red), and G104βP/T96βK (blue). The *K*_D_ values of 868-Z11 T96βK, A94αH/T96βK, and G104βP/T96βK to SL9/HLA-A2 were determined to be 2.5 ± 0.8 µM, 33 ± 9 µM, and 2.2 ± 0.9 µM respectively. Data are reflective of six separate titrations; values are the averages and standard deviations from the six experiments. Note that the fitted curve for the T96βK variant is obscured by that of the G104β/T96βK variant due to its nearly identical affinity.

To characterize the impacts of the A94αH and G104βP mutations, we introduced them separately into 868-Z11 and generated recombinant, single chain mutant and WT 868-Z11 protein. We first measured binding affinities using surface plasmon resonance (SPR). As expected based on prior work with the 868-Z11 TCR (24), the affinities were extremely high, in the single-digit nanomolar range, necessitating the use of a single cycle kinetic approach (30). We measured the *K*_D_ of the A94αH mutant as 5.2 nM and the *K*_D_ of the G104βP mutant as 0.3 nM (**Figure 4A**). Consistent with prior data, the *K*_D_ of WT 868-Z11 was measured as 1.1 nM. Curiously, even though both mutants had positive fitness values, the affinity of the A94αH variant was slightly weaker than WT 868-Z11. While binding affinity is one component of a mutation’s fitness score, the density of each mutant on the yeast cell surface also plays a role and is influenced by features such as protein expression and stability. As the approximately 5-fold change in affinity from WT for A94αH was still within the variances seen in other studies of TCR specificity (9, 31, 32), we proceeded to evaluate both the A94αH and G104βP mutations. Per our hypothesis about the impact of the glycine-to-proline mutation on conformational properties, we found it notable that, although the A94αH mutation impacted both TCR on and off rates, the G104βP mutation only substantially impacted the association rate, with the *k*_on_ increased from approximately 2x10^5^ M^-1^ *s* ^-1^ to 9×10^5^ M^-1^ *s* ^-1^. This suggests the glycine-to-proline mutation may reduce CDR loop fluctuations, maintaining the CDR3β loop in a conformation compatible with the SL9/HLA-A2 ligand.

### 2.3 Introduction of a mutation to bring affinities into a physiological range

Although they facilitated yeast display screening, the single-digit nM affinities of the WT 868-Z11 TCR and the A94αH and G104βP variants are >1000-fold stronger than affinities that typify that typify physiological TCRs and presented technical challenges for assessing specificity biochemically. Moreover, TCRs with such supraphysiological affinities can lead to diminished T cell functions, hindering functional studies and thus broader assessments of specificity (33–35). To address this, we capitalized on the deep mutational scanning data and selected a mutation designed to bring the affinity of the 868-Z11 TCR and the A94αH and G104βP variants into a physiological range. The threonine 96 to lysine mutation in the CDR3β (T96βK) loop had a negative fitness score of -5.7 (**Figure 2**) and was distant from that of both Ala94α and Gly104β (**Figure 3**). Introducing the T96βK mutation reduced the *K*_D_ of WT 868-Z11 approximately 2000-fold to 2.5 μM. Adding the G104βP mutation led resulted in an essentially identical *K*_D_ of 2.2 μM and adding the A94αH mutation led to a weaker *K*_D_ of 33 μM (**Figure 4B**). As these values are within the known range for anti-viral TCRs (36) and were amenable to traditional steady state measurements of binding affinity as well as functional experiments, we proceeded to study how the A94αH and G104βP mutations impact TCR specificity in the background of the T96βK mutation.

### 2.4 The framework mutation enhances TCR specificity as assessed by SL9 viral escape variants

To interrogate the effects of the loop and framework mutations on the specificity of the 868-Z11 TCR, we analyzed seven variants of the SL9 peptide associated with HIV escape from immune recognition. The peptide variants chosen are the most frequently observed according to the Los Alamos HIV sequence database (https://www.hiv.lanl.gov), and include the tyrosine 3 to phenylalanine (Y3F) variant, the valine 6 to isoline (V6I) variant, the threonine 8 to valine variant, as well as all three double mutant combinations and the triple mutant, described previously as the “ultimate escape variant” of SL9 (29, 37). Significant structural data are available for HLA-A2 presenting these variants, and they reflect differences in the displayed peptide surfaces, and in some cases, changes in the peptide backbone (38, 39).

Using steady-state SPR, we determined the affinities of 868-Z11 T96βK and the A94αH/T96βK and G104βP/T96βK variants to each peptide presented by HLA-A2 (**Supplementary Table S2**). This allowed us to rigorously assess specificity through the lens of binding affinity and free energy, and as TCR functional responses *in vitro* generally correlate with binding affinities (9, 32, 40, 41), extrapolate to function. Although the fine details differed as described below, there were three distinct patterns among the peptides studied: compared to the WT SL9 peptide, the Y3F, Y3F/T8V, Y3F/V6I, and Y3F/V6I/T8V peptides weakened TCR binding, the T8V peptide had little impact, and the V6I and V6I/T8V peptides slightly strengthened TCR binding.

**Figure 5:**
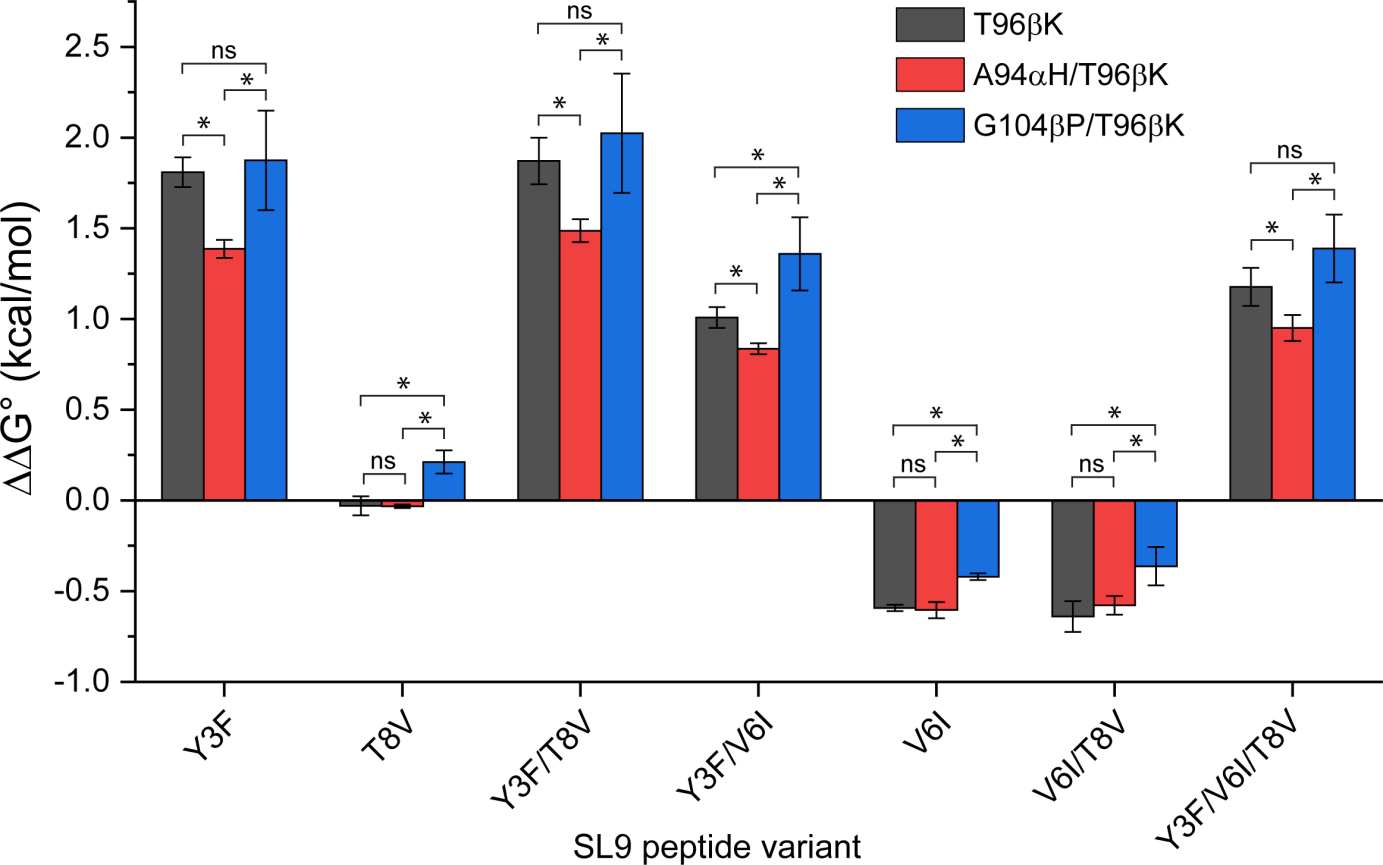
Impacts of mutations in the 868-Z11 TCR on the binding of SL9 escape variants presented by HLA-A2. ΔΔG° values relative to the WT SL9 peptide are shown, determined from steady state *K*_D_ measurements in triplicate. In general, compared to the single T96βK the mutation, addition of the G104βP mutation enhances specificity by shifting ΔΔG° values in a positive (unfavorable) direction, whereas addition of the A94αH mutation weakens specificity by shifting ΔΔG° values in a negative (favorable) direction. ΔΔG° values were determined from three independent *K*_D_ measurements (**Table S2**). Each *K*_D_ was converted to a ΔG° before determining the ΔΔG° relative to WT SL9. The three ΔΔG° values were then averaged, and the standard deviations determined. Statistical differences between mutations were determined using unpaired Student’s *t*-tests (* = significant differences with p< 0.05; ns, differences not significant).

For a biochemically detailed view of specificity, we converted the *K*_D_ values into changes in binding free energy, evaluating ΔΔG° values relative to the WT SL9 peptide (**Figure 5**). The relative ΔΔG° values give an indication of how sensitive each TCR variant is to the escape variants, and thus a direct evaluation of specificity. For all four peptides with the Y3F mutation (Y3F, Y3F/T8V, Y3F/V6I, and Y3F/V6I/T8V), the G104βP/T96βK TCR variant had more unfavorable ΔΔG° values (i.e., the values were more positive). This indicates weaker binding and therefore greater TCR sensitivity to the mutation. For the T8V peptide, the ΔΔG° of the G104βP/T96βK variant was also positive and unfavorable, compared to the negligible values for the T96βK or the A94αH/T96βK variants. For the V6I and V6I/T8V variants where binding was slightly improved compared to the WT SL9 peptide, the improvement was smaller with the G104βP/T96βK variant.

Overall, when compared quantitatively to the T96βK and A94αH/T96βK variants, the more unfavorable ΔΔG° values for the G104βP/T96βK variant reached statistical significance for 4/7 and 7/7 peptides, respectively. In contrast, the A94αH/T96βK variant was more tolerant of the escape variants (i.e., the ΔΔG° values were less positive). Although the ΔΔG° values are still small, a defining characteristic of T cell biology is the amplification of very small changes in binding affinity into large functional differences (42). Thus, as assessed by the SL9 escape variants, the framework G104βP mutation enhanced specificity, while the interfacial A94αH mutation weakened specificity.

### 2.5 The framework mutation enhances TCR specificity as assessed by a positional scanning peptide library

To gain a broader, functional view of TCR specificity, we assessed TCR recognition using a positional scanning library of the SL9 peptide, which varies each amino acid of the peptide with all 20 standard amino acids, excluding primary anchors. Positional scanning libraries (sometimes referred to as X-scans) are frequently used to assess the specificity of TCR clinical candidates (43, 44). Although they are less quantitative than binding or functional titrations, they permit the rapid assessment of a wider array of peptides. The library scan was restricted to the 868-Z11 G104βP/T96βK and T96βK variants, as the two TCR variants bound with essentially identical affinities, facilitating a direct comparison.

**Figure 6:**
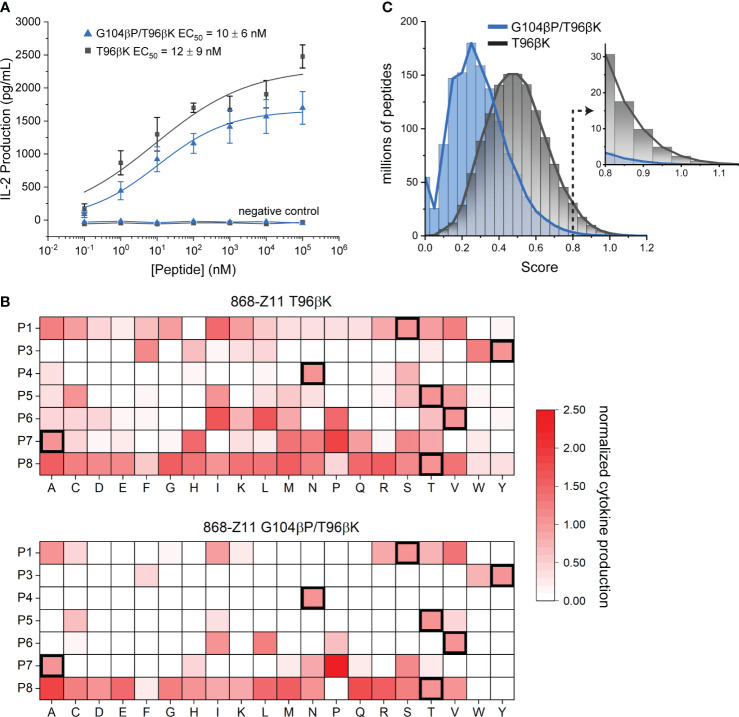
Positional scanning library analysis confirms the G104βP mutation confers higher specificity. **(A)** The T96βK and G104βP/T96βK variants of 868-Z11 have EC_50_ values identical within error for the SL9 peptide in functional assays measuring cytokine release. Data points are averages and standard deviations of five separate titrations; values reported are the averages and standard deviations from the five experiments. Negative control data are for co-cultures with the irrelevant Tax_11-19_ peptide (sequence LLFGYPVYV). **(B)** Positional scanning library data for the T96βK and G104βP/T96βK variants of 868-Z11. For each peptide in the library, IL-2 production at 10 µM peptide in a co-culture experiment is normalized to that of the WT SL9 peptide as indicated by the scale on the right. Addition of the G104βP mutation results in fewer stimulatory peptides, particularly in the C-terminal half of the peptide. Data in each cell are the average of three separate co-culture experiments. **(C)** Fingerprint analysis from the data in panel B, showing the distribution of scores for all 1.28 billion peptides of the form XLXXXXXXL, where X is any of the 20 standard amino acids. The greater specificity conferred by the G104βP mutation is indicated by the left-shifted blue curve, further highlighted by the much smaller number of peptides with scores ≥ 0.8 as indicated by the inset.

We first separately transduced full length 868-Z11 G104βP/T96βK and T96βK TCRs into CD8+ Jurkat 76 cells (45), which do not produce TCR in the absence of transduced α and β chains. We incorporated CD8 to strengthen the signal as well as better mimic T cell biology. After selecting and expanding high TCR expressing transfectants (**Supplementary Figure S2**), cells were co-cultured with TAP-deficient HLA-A2+ T2 cells with increasing concentrations of the WT SL9 peptide. IL-2 produced from the co-culture was measured by ELISA in order to determine peptide EC_50_ values. Although maximum amounts of IL-2 produced differed due to slight differences in transfection efficiencies or possibly protein stability, the EC_50_ values from these experiments were identical within error, with values of 12 nM for T96βK and 10 nM for G104βP/T96βK (**Figure 6A**). This result is consistent with the variants’ identical binding affinities, indicating that, in this case, the recognition behavior of the TCR biological assembly matches that of the recombinant protein, and supports a head-to-head comparison of the two TCRs using the SL9 positional scanning library.

We next performed co-culture experiments with individual peptides from the positional scanning library. We selected a peptide concentration of 10 μM to ensure responses from weakly stimulatory peptides were captured, but still below the saturating concentration, as we expected some peptides could be more potent than WT SL9. The library results confirmed the higher specificity of the G104βP/T96βK variant, as compared to the T96βK variant, fewer peptides elicited responses (53 for G104βP/T96βK vs. 119 for T96βK), and of those that did, fewer reached the level of WT or above (19 for G104βP/T96βK vs. 30 for T96βK) (**Figure 6B**). Notably, while the higher specificity of the G104βP/T96βK variant was seen at every position of the peptide, it was most obvious in the C-terminal half, which has the greatest contact with the CDR3β loop (**Figure 3**).

We next used the data to generate theoretical TCR “fingerprints” that score all possible 1.28 billion (20^7^) combinatorial peptide variants (46, 47). The IL-2 response of each amino acid at each position was normalized to the IL-2 response of the WT peptide. For any theoretical peptide, the normalized responses for each amino acid were summed and divided by 7, the sum of the normalized responses of the amino acids of the WT SL9 peptide. TCR fingerprints make the limiting assumption that every substitution at each peptide position acts independently of others, an assumption that can be violated through compensatory or cooperative substitutions (48). This assumption notwithstanding, fingerprints still allow a theoretical comparison between TCRs and provide a high-level window into overall specificity.

The differences in the positional scanning library responses were reflected in the fingerprint analysis, with the values for the G104βP/T96βK variant downshifted compared to those of the T96βK variant (i.e., more peptides received lower, weakly/non-stimulatory scores) (**Figure 6C**). In previous work, we identified peptides with TCR fingerprint scores of 0.8 or higher as those more likely to lead to some level of functional recognition, with higher scores associated with higher binding affinity and stronger responses (47). The T96βK variant had approximately 67 million peptides in the range of 0.8 and above, while G104βP/T96βK had approximately 10-fold fewer (6.6 million) (**Figure 6C**, inset). For another window into specificity, we tabulated the number of peptides with up to three amino substitutions found in the scores of 0.8 or higher, choosing three substitutions to reflect the maximum number seen in the SL9 escape variants, and recognizing that peptides with four or more substitutions are likely to have significantly altered conformations in the groove. The G104βP/T96βK variant had 43,163 peptides with up to three mutations with scores of 0.8 or higher. In contrast, the less specific T96βK variant had 112,317 such peptides, almost three times as many.

### 2.6 Molecular dynamics simulations indicate enhanced specificity emerges from reduced loop flexibility

To gain insight into how the G104βP mutation enhances the specificity of the 868-Z11 TCR, we studied protein flexibility using molecular dynamics simulations. Using the structure of the 868 TCR bound to SL9/HLA-A2 as a template (29), we introduced mutations to generate the 868-Z11 T96βK, A94αH/T96βK, and G104βP/T96βK TCRs. After energy minimization and equilibration, each of the free TCRs (i.e., not bound to SL9/HLA-A2) were simulated in explicit solvent for 1 μs. From these trajectories we examined root mean square (RMS) fluctuations of the CDR loops of both the α and β chains.

**Figure 7:**
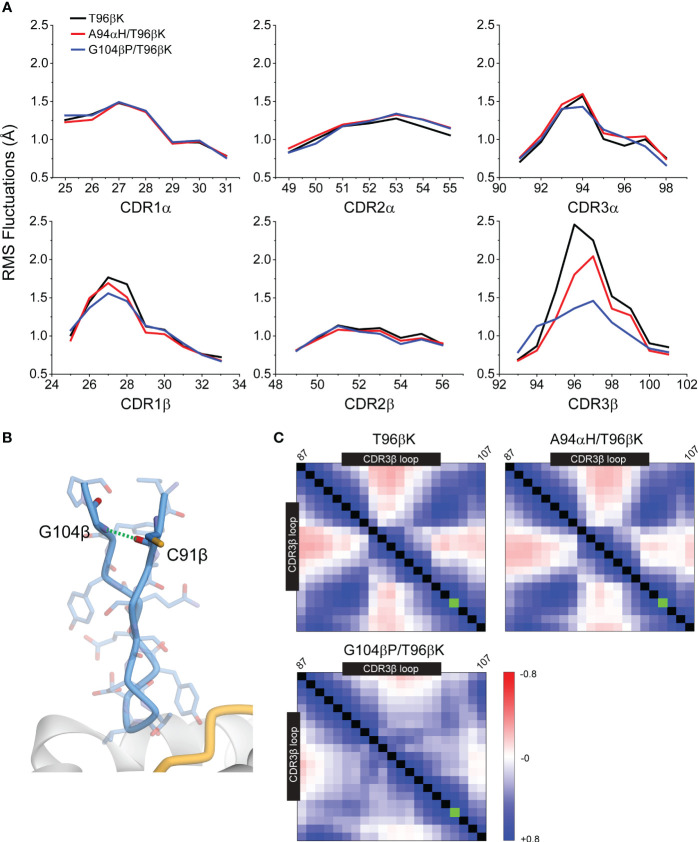
The G104βP mutation reduces the magnitude of fluctuations in the 868-Z11 TCR CDR3β loop. **(A)** The fluctuations for all six loops of the α and β chains for the T96βK, A94αH/T96βK, and G104βP/T96βK 868-Z11 TCR variants determined from 1 μs of molecular dynamics simulations of the unbound TCR. The largest reduction with the G104βP mutation is seen in the CDR3β loop. **(B)** Structural view of the 868-Z11 CDR3β loop and the framework mutations above it, highlighting the backbone hydrogen bond between Gly104 and Cys91 that is lost with the glycine-to-proline mutation. **(C)** Cα motional cross-correlation matrices for the CDR3β loop and the framework amino acids above it (residues 87-107) from the molecular dynamics simulations described in panel **(A)**. Amino acids of the loop are indicated by the black boxes. The green box indicates position 104β. As indicated by the scale, blue is positively correlated motion, red is negatively (anti) correlated motion. Addition of the G104βP mutation, but not the A94αH mutation, substantially reduces the magnitude of correlated motion between the center of the loop and the residues above it.

The RMS fluctuation data indicated that the CDR3β loop possessed the highest flexibility (**Figure 7A**). The A94αH mutation had only a small impact on the fluctuations of this loop, reducing RMS fluctuations at the center by approximately 0.4 Å. In contrast, the G104βP mutation led to a marked reduction in CDR3β fluctuations, reducing fluctuations at the loop center by approximately 1 Å. The G104βP mutation also slightly impacted the fluctuations in CDR1β and CDR3α, reducing movement at the loop centers by approximately 0.3 Å and 0.2 Å, respectively. Reduced fluctuations are consistent with the conclusion that the glycine-to-proline substitution in the framework region limits the ability of the CDR3β and, to a lesser extent its adjacent loops, to structurally adjust to different ligands, thereby enhancing TCR specificity. This conclusion is also consistent with the impact of the G104βP mutation on the association rate for TCR binding, which as noted above was faster than either the WT 868-Z11 TCR or the variant with the A94αH mutation (**Figure 4A**).

The G104βP mutation did not reduce loop flexibility by altering backbone torsion distributions, as ϕ/ψ bond distributions at positions 104 and 105 were the same in all three simulations (**Supplementary Figure S3**). Rather, with glycine at position 104β, an interstrand backbone (NH to CO) hydrogen bond is formed to Cys91 above the N-terminal end of the CDR3β loop (**Figure 7B**). Removing this hydrogen bond by replacing glycine with a proline reduced correlated motion within the loop, most notably between the loop apex and the ends of the loop as they enter the framework (**Figure 7C**). With less correlated motion, overall en bloc motions of the loop are reduced, allowing thermal fluctuations to occur more independently and ultimately leading to smaller magnitude motions at the apex, as seen by the RMSF analysis. The reduction of correlated motion without reduced intrinsic dynamics provides an explanation for how the mutation alters loop properties without altering binding affinity towards SL9/HLA-A2, as otherwise the mutation would be expected to strengthen binding by reducing entropic penalties.

We next examined the conservation of the interstrand glycine-to-cysteine hydrogen bond above CDR3β in TCRs. In 558 structures representing 260 unique TCRs, this hydrogen bond is present in 540 (97%). To ask if the glycine-to-proline mutation in CDR3β might have similar effects on loop dynamics with other TCRs, we simulated the A6 and LC13 TCRs with and without the glycine-to-proline mutation. The CDR3β loops of both the A6 and LC13 TCRs, which are divergent from 868, undergo conformational changes upon binding peptide/MHC (49–52), and molecular dynamics simulations have characterized the high frequency motions of the CDR3β loops of both receptors (49, 53, 54). In 1 μs molecular dynamics simulations performed identically as those for the 868 variants, the mutation of glycine to proline had differing effects on the CDR3β loops of two TCRs, with a small reduction in RMS fluctuations for A6 and little to no impact on the LC13 (**Supplementary Figure S4**). Thus, although the glycine-to-cysteine interstrand hydrogen bond is conserved across TCRs, its removal through mutation is not predicted to have conserved consequences for loop motions, indicating that sequence variations within different loops will likely impact the level of control framework regions have over motional properties. Lastly, we queried the frequency of this mutation in known antibody sequences. As noted above, antibodies share an analogous pre-CDR3 sequence, and the same glycine to proline mutation studied here was identified in a broadly neutralizing antibody to HIV gp120 (20, 22). Using the Observed Antibody Space database (55), in approximately 7 million sequences, we found 1203 instances of a glycine to proline mutation above antibody CDR3 loops, indicating that the mutation is rare (as expected for a framework mutation) but still potentially impactful as seen with HIV gp120.

## 3 Discussion

With ongoing efforts to develop TCR and T-cell based therapeutics, interest has grown in improving TCR specificity through protein engineering. Unlike antibodies, however, enhancing TCR affinity generally does not improve specificity. This can be attributed to the structural features of the TCR’s composite ligand, which presents the generally flat target peptide within a structurally conserved MHC platform, as well as the vast universe of MHC-presented peptides, which ensures that many structurally and chemically similar targets are regularly encountered. With a handful of notable outliers [e.g., refs (56, 57)]. T cell functional responses generally correlate with the binding affinity of the TCR (9, 32, 40, 41). As T cell responses can be elicited over a range of relatively weak TCR binding affinities (generally, *K*_D_’s ranging from single digit to hundreds of micromolar), enhancing TCR affinity towards one peptide is very likely to enhance affinity towards others, thereby bringing otherwise non-stimulatory off-target peptides into a stimulatory range (9). This challenge has led to a variety of efforts to improve TCR specificity through protein engineering efforts, including the use of negative design principles as well as the development of non-TCR based peptide/MHC recognition molecules such as antibodies or other proteins (58, 59).

The CDR loops of TCRs, and the hypervariable CDR3 loops in particular, often exhibit a degree of flexibility, contributing to the cross-reactivity that is necessary for a T cell repertoire to respond to a much larger pool of possible antigens (2, 3, 60). This is true for the 868 TCR studied here, whose CDR3α and CDR3β loops undergo large structural changes upon binding the SL9 epitope presented by HLA-A2 (29). Conformational changes in CDR loops, which occur over a range of timescales and reflect both conformational selection as well as induced-fit (49, 61–66), afford the opportunity to impact TCR binding though mutations that alter loop motions. Particularly attractive are mutations in framework regions outside of the traditionally defined CDR loops that are at or near hinge regions, as these would not be expected to directly alter a TCR’s structural and chemical fit with different peptides and thus inadvertently introduce new reactivities. Framework mutations have indeed been shown to impact TCR binding affinity towards specific ligands (16). Importantly though, as they do not directly alter the contact surface, mutations in these positions should also be able to alter or even improve TCR specificity. Indeed, naturally selected as well as engineered framework mutations are associated with the generation of more potent and specific antibodies (20–23).

Here we demonstrated how framework vs. interface mutations in the 868-Z11 TCR impacted specificity towards the HIV SL9 epitope presented by HLA-A2. In the background of a mutation to bring binding affinity into a physiological range, neither mutation greatly impacted affinity towards SL9/HLA-A2, permitting a direct comparison of specificity. Tested with a range of SL9 escape variants, the framework mutation – a glycine to proline mutation above CDR3β – indeed led to improved specificity. This was first apparent with escape variants of the SL9 peptide assessed biochemically. Although the effects on binding were subtle, T cells via their signaling mechanisms magnify small differences in binding affinity (42). This was clear when we examined T cell responses to a positional scanning library of the SL9 peptide, where the framework mutation led to substantially improved specificity. On the other hand, the interfacial alanine to histidine mutation near the apex of CDR3α directly changed the TCR contact surface. Although the mutation did not impact binding to the SL9 epitope, this variant better tolerated SL9 peptide variants, revealing a loss of specificity.

The mechanism of improved specificity for the glycine to proline mutation is of interest, as it does not appear to result directly from limitations on backbone torsions that might be expected by changing a glycine to a proline. Rather, the effect results from the loss of a hydrogen bond across the framework above the CDR3β loop, which reduces correlated motion, reducing the overall magnitude of loop fluctuations. Intriguingly, an analogous proline mutation above CDRH2 that also eliminated an inter-strand hydrogen bond was identified in a broadly neutralizing antibody to HIV gp120; reversion back to the native alanine reduced neutralization potency against several strains (20, 22). Alterations to hydrogen bonds through proline mutations have had similar dynamic effects in other systems (67). Although altering hydrogen bonds at or near the start of CDR loops may be a strategy for controlling TCR loop dynamics and thus specificity, our simulations with other TCRs suggest that unique features of CDR loops will also have an impact, likely necessitating a screening approach as applied here. Nonetheless, our overall results indicate that manipulation of TCR framework regions may be a productive approach for sharpening the specificity of clinical candidates and thus reducing the risk of unanticipated off-target recognition in TCR-based therapies. Careful engineering could similarly be used to engineer TCRs that target a range of related peptides, mimicking in principle the “multi-specific” TCRs that recognize similar tumor associated antigens (68).

## 4 Experimental procedures

### 4.1 Preparation of mutagenesis libraries

The gene for the 868-Z11 scTCR was inserted into the pETconNK plasmid (Addgene #81169) using the *NdeI* and *XhoI* restriction sites. Comprehensive single site saturation mutagenesis libraries of the 868-Z11 scTCR in the pETconNK plasmid were constructed using nicking mutagenesis as described previously (69). Mutagenic oligos were obtained from Integrated DNA Technologies. Two separate libraries were generated: library 1 covered mutations in the α chain and library 2 covered mutations in the β chain. Library plasmid DNA was transformed into chemically competent *S. cerevisiae* EBY100, grown, and stored in yeast storage buffer (20% w/v glycerol, 20 mM HEPES, and 150 mM NaCl pH 7.5) at -80°C according to published protocols (70).

### 4.2 Screening of 868-Z11 libraries

To screen the 868-Z11 single site saturation mutagenesis libraries, 1×10^7^ cells were grown from freezer stocks in 1 mL of SDCAA (2% w/v dextrose (D-glucose), 0.67% w/v yeast nitrogen base without amino acids, 0.5% w/v Bacto casamino acids technical, 0.54% w/v Na_2_HPO_4_, and 0.856% w/v Na_2_HPO_4_·H_2_O) for 6 hours at 30°C and reinoculated at OD_600_ = 1.0 in 1 mL of induction media, SGCAA at 18°C for 16 hours. For each library, 2×10^7^ yeast were labeled with SL9/HLA-A2 tetramers at a concentration of 1 nM for 30 min at 4°C (reduced temperature to help ensure stability and viability) in PBS-BSA (pH 7.4: 0.8 w/v NaCl, 0.02% w/v KCl, 0.144% w/v Na_2_HPO_4_, and 0.024% w/v KH_2_PO_4_, 0.1% w/v bovine serum albumin). After centrifugation at 2500*g* for 5 minutes, removal of supernatant, and resuspension in 1.95 mL of PBS-BSA, cells were secondarily labeled with 48 μL of anti-c-Myc-FITC (Miltenyi Biotec 130-116-485, lot number 5200301418) for 10 min at 4°C. Before sorting, the cells were washed with 5 mL of PBS-BSA, centrifuged at 2500*g*, the supernatant was removed, and cells were resuspended in PBS-BSA immediately before sorting with a Sony SH800S instrument. Each sort collected approximately 100-fold of the theoretical diversity at the amino acid level using a gate set to collect the top 25% of the double positive population. A reference population for each library was collected from the “single cell” gated population. Collected cells from each population were recovered at 30°C for 30 hours in 10 mL of SDCAA, washed with PBS-BSA, and then stored in 1 mL of yeast storage buffer at a concentration of 4×10^7^ cells per mL at -80°C. Libraries were prepared for deep sequencing as previously described (70). The libraries were pooled and sequenced on an Illumina MiSeq using 2×250 bp pair-end reads at the BioFrontiers Sequencing Facility at CU Boulder.

### 4.3 Deep mutational scanning data analysis

The Protein Analysis and Classifier Toolkit (PACT) (modified version of Enrich 2) (28) was used to compute enrichment ratios from the raw sequencing files. Python scripts available at Github (user: JKlesmith) were used to normalize the enrichment ratios *E_i_* from:


$$E_{i}=log_{2}\left(\frac{f_{i,sel}}{f_{i,ref}}\right)$$

where *f_i,sel_* is the frequency of variant *i* in the selected population, and *f_i,ref_* is the frequency of variant *i* in the reference population. For comparison between libraries, a fitness score (*F_i_*) for each mutant *i* was calculated as:


$$F_{i}=E_{i}-\;E_{wt}$$

where *E_i_* is the enrichment ratio of the selected mutant and *E_wt_* is the enrichment ratio of the wildtype.

### 4.4 Yeast display affinity titration

Yeast expressing the 868-Z11 scTCR were induced and grown to an OD_600_ of 2. For each point in the titration, 5 µL cells were incubated with SL9/HLA-A2 tetramer at concentrations ranging from 256 pM to 33 nM for 30 min at 4°C (lower temperature chosen to help ensure stability and viability). Cells were centrifuged at 2500*g* for 5 minutes, washed with 5 mL PBS-BSA, and resuspended in PBS-BSA before analysis on a Sony SH800S instrument. The mean fluorescent intensity (MFI) as a function of tetramer concentration was utilized to determine the apparent binding affinity using a 1:1 binding model.

### 4.5 Protein expression and inclusion body purification

Genes encoding soluble constructs for the HLA-A*02:01 (HLA-A2) heavy chain, β_2_-microglobulin (β_2_m), and the 868-Z11 scTCR in pET28 vector were purchased from Genewiz. Recombinant proteins were overexpressed in *Escherichia coli* as inclusion bodies under the control of an IPTG-inducible T7 promoter. Individual proteins were expressed in either BL21 (DE3) *E. coli* or Rosetta (DE3) *E. coli* after inducing cultures with 0.5- 1.0 M IPTG at an OD_600_ > 0.5. Once induced, cultures were grown for 6-8 hours and harvested at 9000 rpm. Collected cells were resuspended in 50 mM Tris-HCl, 1mM EDTA, 250 g/L sucrose, 1 g/L sodium azide, and 10 mM DTT. Subsequently, cells were lysed with lysis buffer (50 mM Tris-HCl, 100 mM NaCl, 1% Triton X-100, 10 g/L sodium deoxycholate, 1 g/L sodium azide, and 10 mM DTT), 50 mg lysozyme, 2 mg DNase (Worthington), and 5 mM MgCl_2_. Next, 10 mM EDTA was added, and the cell lysate sonicated. Inclusion bodies were then isolated from the cell lysate by centrifugation at 9000 rpm. Once isolated, inclusion bodies were washed with an initial wash buffer (50 mM Tris-HCl, 1 mM EDTA, 100 mM NaCl, 0.5% Triton-X 100, 1 g/L sodium azide, and 125 mM DTT) and then final wash buffer (50 mM Tris-HCl, 1 mM EDTA, 100 mM NaCl, 1g/L sodium azide, and 125 mM DTT). The inclusion bodies were solubilized in final buffer (8 M urea, 25 mM MES pH 6.0, and 1 mM DTT) before centrifugation at 17,000 rpm to remove any remaining insoluble debris and stored at -80°C until use. The concentration of the solubilized inclusion bodies was determined with a Bradford assay. Peptides were commercially synthesized from AAPPTEC or GenScript at >80% purity and diluted to 30 mM in DMSO.

### 4.6 Protein production and purification

Inclusion bodies were diluted with 6 M guanidinium-HCl, 10 mM EDTA, 10 mM sodium acetate pH 4.2 and incubated at 37°C prior to refolding. For refolding of all soluble, recombinant HLA-A2 complexes, inclusion bodies of β_2_m (30 mg/L) and peptide (30 μM) were first added to refolding buffer consisting of 400 mM L-arginine, 2 mM EDTA, 100 mM Tris-HCl pH 8.3, 6.3 mM cysteamine, 3.7 mM cystamine, and 0.2 mM PMSF at 4°C. After 1 hour of incubation, inclusion bodies of HLA-A2 heavy chain (30 mg/L) were added to the refolding buffer, β_2_m, and peptide. For soluble, recombinant scTCRs, 30 mg/L of inclusion bodies were added to refolding buffer consisting of 2 M guanidinium-HCl, 2 mM EDTA, 50 mM Tris-HCl pH 8.3, 6.5 mM cysteamine, 3.7 mM cystamine, and 0.2 mM PMSF. The refolded proteins were then purified via DEAE anion-exchange chromatography (Whatman/GE Healthcare) followed by size-exclusion chromatography on a HiLoad Superdex 200 column (GE Healthcare) followed by HiLoad Superdex 75 column (GE Healthcare). Protein concentrations were determined through UV absorbance using sequence-determined extinction coefficients from EXPASY ProtParam.

### 4.7 Surface plasmon resonance

Surface plasmon resonance was performed on a Biacore T200 instrument. All proteins were purified in HBS-EP (10 mM HEPES, 150 mM NaCl, 3 mM EDTA, and 0.005% surfactant P-20, pH 7.4). For analysis, scTCR was immobilized on a CM5 Series S sensor chip to approximately 2000 RU for steady state analysis and 100-300 RU for single cycle kinetics via amine coupling. For steady state measurements, peptide/MHC was injected at a flow rate of 5 μL/min at concentrations between 50 µM and 2 nM, while single cycle kinetics experiments were performed at a flow rate of 100 μL/min with injected concentrations ranging from 0.6 – 48 nM. All experiments were performed at 25°C with a blank activated/deactivated flow cell as a reference. Data were evaluated in BiaEvaluation 4.1 before being fit to a 1:1 binding model in OriginPro 2019 or BiaEvaluation 4.1 for steady state and single cycle kinetics experiments, respectively. Statistical tests of binding data were determined using OriginPro 2019.

### 4.8 Peptide/MHC tetramer production

To produce peptide/MHC tetramers for staining of yeast libraries, a BirA substrate biotinylation tag was added to the C-terminus of the HLA-A2 heavy chain and expressed, refolded, and purified as described above. Once purified, SL9/HLA-A2 was buffer exchanged into 50 mM bicine pH 8.3 at which point the biotinylation reagents and BirA enzyme were added (41 μM peptide/MHC, 480 μM D-biotin, 0.48 μM Bir A enzyme, 5 mM MgCl_2_, and 2 mM ATP) and incubated at room temperature while mixing for 4 hours. Next, 12 μL of HisPur cobalt resin (Thermo Scientific) and 500 μL of 50 mM bicine, 25 mM imidazole pH 8.3 was added and the reaction was allowed to incubate for another hour at room temperature. After incubation, the HisPure cobalt resin was pelleted by spinning at maximum speed, and the supernatant containing the biotinylated pMHC was removed and purified on a HiLoad Superdex 75 column. To generate peptide/MHC tetramer, streptavidin-phycoerythrin (SAPE) was added in a 1:4 SAPE:peptide/MHC molar ratio to peptide/MHC in four separate additions with a 10 minute incubation period between additions.

### 4.9 Generation and maintenance of TCR-expressing Jurkats and T2 antigen presenting cells

CD8+ Jurkat 76 and T2 cell lines were maintained in PRMI-1640 media supplemented with 10% fetal bovine serum (FBS), 100 units/mL penicillin, and 100 μg/mL streptomycin. To generate stable TCR expressing cell lines, CD8^+^/CD34^-^ Jurkat cells were transduced using the Neon transfection System (Thermo Fisher) with the pCMV(CAT)T7-SB100 plasmid and the pSBbi-Neo Sleeping Beauty vector encoding full length TCR α and β chains separated by the P2A self-cleaving peptide. Transformants were positively selected by culturing in complete media containing 1.2 mg/mL of G418. Flow cytometry was used to analyze TCR expression and select high TCR expressing transformants using a BD FACSMelody instrument. For selection, transduced cells were stained with anti-human CD8 FITC conjugated antibody (SK1) (BioLegend 344704, lot number B367025) and anti-human CD3 APC/Cy7 conjugated antibody (UCHT1) (BioLegend 300426, lot number B378992) and the top 50% of CD3+ cells selected.

### 4.10 T cell co-culture experiments

For all co-culture experiments (titration with the WT SL9 peptide as well as positional scanning libraries), 1×10^5^ T2 cells were pulsed with peptides (10 µM for PSL and 0.1 nM-100 µM for titrations) for 2 hours at 37°C. After peptide pulsing, 1×10^5^ TCR-expressing CD8+ Jurkat76 cells incubated with 50 ng/mL phorbol 12-myristate 13-acetate for approximately 1 hour were added to the T2 cells and the co-culture allowed to incubate for 18-20 hours at 37°C (71, 72). IL-2 release was measured by ELISA (BioLegend 431816). The EC_50_ titrations were repeated five times and each dataset fit separately, and the resulting values averaged. The library experiments were repeated three times and the values averaged.

### 4.11 Positional scanning library analysis

IL-2 values in the PSL co-cultures were used to generate a position weight matrix as performed previously (46, 47), except that, based on the IL-2 production data, peptides potentially more potent than the WT SL9 peptide were permitted to score higher. All values for each cell in the PSL heat map were normalized by the IL-2 response for the WT SL9 peptide to generate the matrix. Scores for any theoretical peptide matching the motif XLXXXXXXL, where X is any one of the 20 standard amino acids, were generating by summing the normalized value for each amino acid at that position and dividing by the sum of the values for the WT peptide (equal to 7). The resulting scores for the 1.28 billion peptides (20^7^) were binned with a size of 0.05.

### 4.12 Molecular dynamics simulations and structure analysis

Molecular dynamics simulations were performed as previously described (73, 74). Briefly, simulations were conducted using the Amber18 molecular dynamics package with GPU acceleration (75). For the 868 TCR, initial coordinates were obtained from PDB deposition 5NME utilizing the coordinates of the TCR only (29). For A6 and LC13, initial coordinates were obtained from PDB depositions 3QH3 and 1KGC, respectively (49, 50). For A6, molecular dynamics simulations were performed utilizing the coordinates of the first molecule in the asymmetric unit. Mutations (T96βK, A94αH, G104βP, and Z11 conversion mutations for 868-Z11, G107βP and G109βP for A6 and LC13, respectively) were incorporated via the “swapaa” command in UCSF Chimera (76). Protonation states for ionizable side chains were determined by the APBS-PDB2PQR web service at pH 7.0 (77). All simulations were performed using the ff14SB forcefield (78). Each structure was solvated in a cubic box of SPC/E water and charge neutralized with sodium. Structures were minimized and then gradually heated to 300 K in the NVT ensemble using a Langevin thermostat while employing solute restraints. Solute restraints were slowly relaxed from 25 kcal/mol/Å^2^ to 0 kcal/mol/Å^2^, and each system was subsequently equilibrated for 10 ns in the NPT ensemble. Production simulations were then calculated in the NVT ensemble utilizing a 2 fs time step and an 8 Å cutoff for nonbonded interactions, all while employing the SHAKE algorithm to restrain bonds involving hydrogen (79). Production simulations were calculated for a total time of 1 μs. Analyses of all trajectories were performed with cpptraj (80). RMS fluctuations were calculated via the “atomicfluct” command following global Cα superimposition and 2D dynamical cross-correlation matrices were calculated via the “matrix correl” command. Visualization and structure analysis was performed with Chimera, PyMOL, and Discovery Studio. Interstrand hydrogen bonds in TCR structures were identified via the “hbonds” command in UCSF Chimera (76), utilizing TCRs listed within the TCR3d database current as of November 8, 2023 (81).

## Data availability statement

The raw data supporting the conclusions of this article will be made available by the authors, without undue reservation. Data are available at the Zenodo archive with DOI 10.5281/zenodo.10685013 (https://zenodo.org/records/10685013).

## Author contributions

AR: Conceptualization, Data curation, Formal analysis, Investigation, Validation, Writing – original draft, Writing – review & editing. CA: Conceptualization, Data curation, Formal analysis, Investigation, Visualization, Writing – review & editing. AM-C: Investigation, Methodology, Resources, Writing – review & editing. TW: Funding acquisition, Methodology, Resources, Supervision, Writing – review & editing. BB: Conceptualization, Funding acquisition, Project administration, Supervision, Visualization, Writing – original draft, Writing – review & editing.
