# Supplementary material for: Enhanced T cell receptor specificity through framework engineering
Source: Front Immunol. 2024 Mar 12;15:1345368. doi: 10.3389/fimmu.2024.1345368 (PMC10967027; doi:10.3389/fimmu.2024.1345368)
Supplement: Supplementary file 1 [file DataSheet_1.pdf]

Supplementary Material

	Library 1 – α chain	Library 2 – β chain
Total number of reads:	241,078	440,789
Reference population reads with		
No nonsynonymous mutations:	118,349 (49.1%)	225,660 (51.2%)
Nonsynonymous mutations:	113,562 (47.1%)	205,427 (46.6%)
Multiple nonsynonymous	9167 (3.8%)	9702 (2.2%)
Coverage of single nonsynonymous amino acid mutations:	778/780 (99.7%)	839/840 (99.9%)

Supplementary Table S1. Statistics of deep mutational scanning yeast libraries for the 868-Z11 TCR. Reference populations of each yeast library were collected from the single cell gate (Fig. S1) and deep sequenced. Library 1 encompasses all mutations within the α chain of the 868-Z11 TCR and library 2 encompasses all mutations within the β chain. The libraries incorporated all but three mutations of interest (99.8%).

Group 1

	SL9	SL9 Y3F	SL9 T8V	SL9 Y3F/T8V	SL9 Y3F/V6I
**T96βK**	1.6	38.6	1.6	46.0	9.6
	1.9	39.3	1.9	44.6	10.2
	3.1	58.2	2.7	59.0	15.7
**A94αH / T96βK**	23.4	263.7	22.2	323.9	102.1
	26.1	274.4	24.3	313.6	104.9
	42.3	403.1	40.7	474.7	167.2
**G104βP / T96βK**	0.9	34.0	1.4	49.1	12.5
	2.5	39.8	3.5	49.1	18.5
	2.5	52.3	3.2	62.5	22.5

Group 2

	SL9	SL9 V6I	SL9 V6I/T8V	SL9 Y3F/V6I/T8V
**T96βK**	2.0	0.8	0.8	17.3
	3.2	1.1	1.1	23.8
	3.4	1.2	1.0	20.2
**A94αH / T96βK**	28.7	9.5	9.9	154.5
	33.4	12.4	13.7	176.7
	47.0	17.9	17.9	203.3
**G104βP / T96βK**	1.4	0.7	0.8	20.4
	2.9	1.5	1.8	28.1
	3.3	1.6	1.5	26.3

Supplementary Table S2. Affinity measurements for 868-Z11 T96βK, A94αH/T96βK, and G104βP/T96βK binding to SL9/HLA-A2 and SL9 escape peptides determined through steady state SPR at 25 °C. Values are K_D's in μM. The peptides were broken into two groups and measured in triplicate, with independent WT measurements for each group.

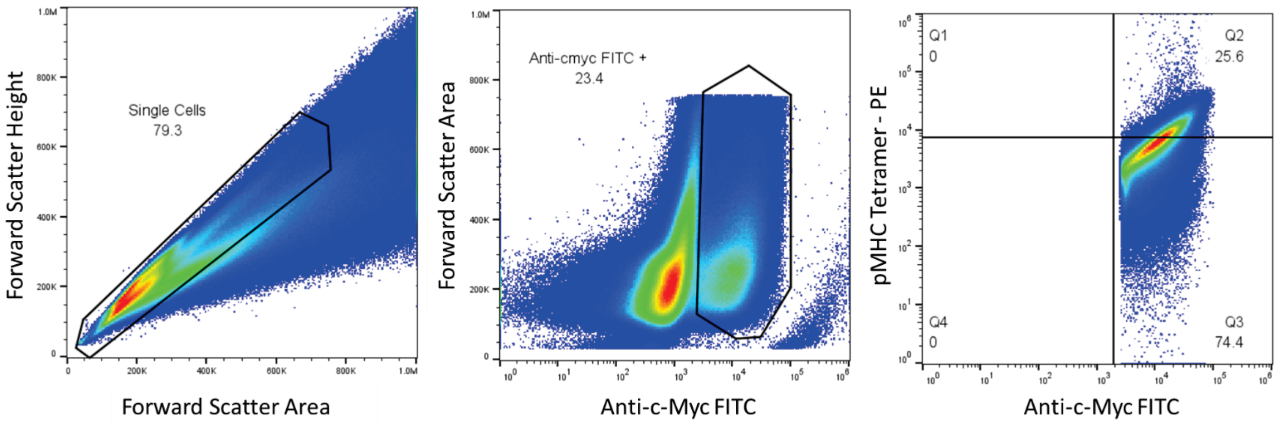

Supplementary Figure S1. Representative flow cytometry gating of deep mutational scanning yeast display libraries. Yeast display libraries were stained with peptide/MHC tetramer and anti-c-Myc antibody before sorting. The libraries were first gated for single cells, followed by a c-Myc+ gate, and then for the top 25% of tetramer positive cells. The reference population was isolated from the single cell population while the selected cells were isolated from the top 25% of tetramer positive cells.

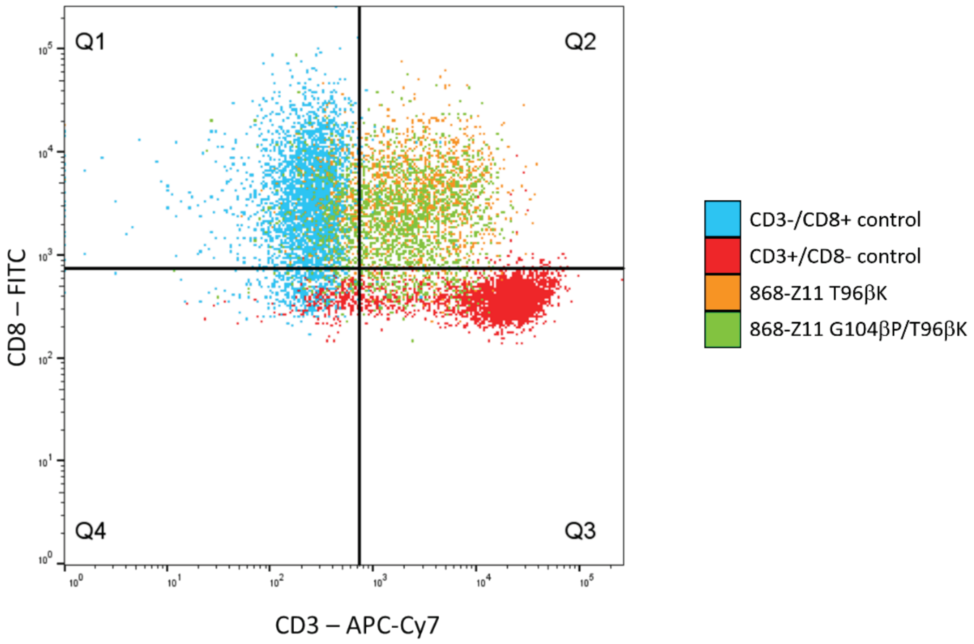

Supplementary Figure S2. Flow cytometry analysis of TCR expressing Jurkat76 cells for co-culture experiments. Flow cytometry analysis of transfected CD8+ J76 868-Z11 T96βK and G104βP/T96βK cells. Cells were stained with anti-CD3 and anti-CD8 antibodies. Two control cell lines were utilized to determine the gates for CD3+ (non-transfected CD8+ J76 cells) and CD8+ (TCR transfected CD8- J76 cells) populations.

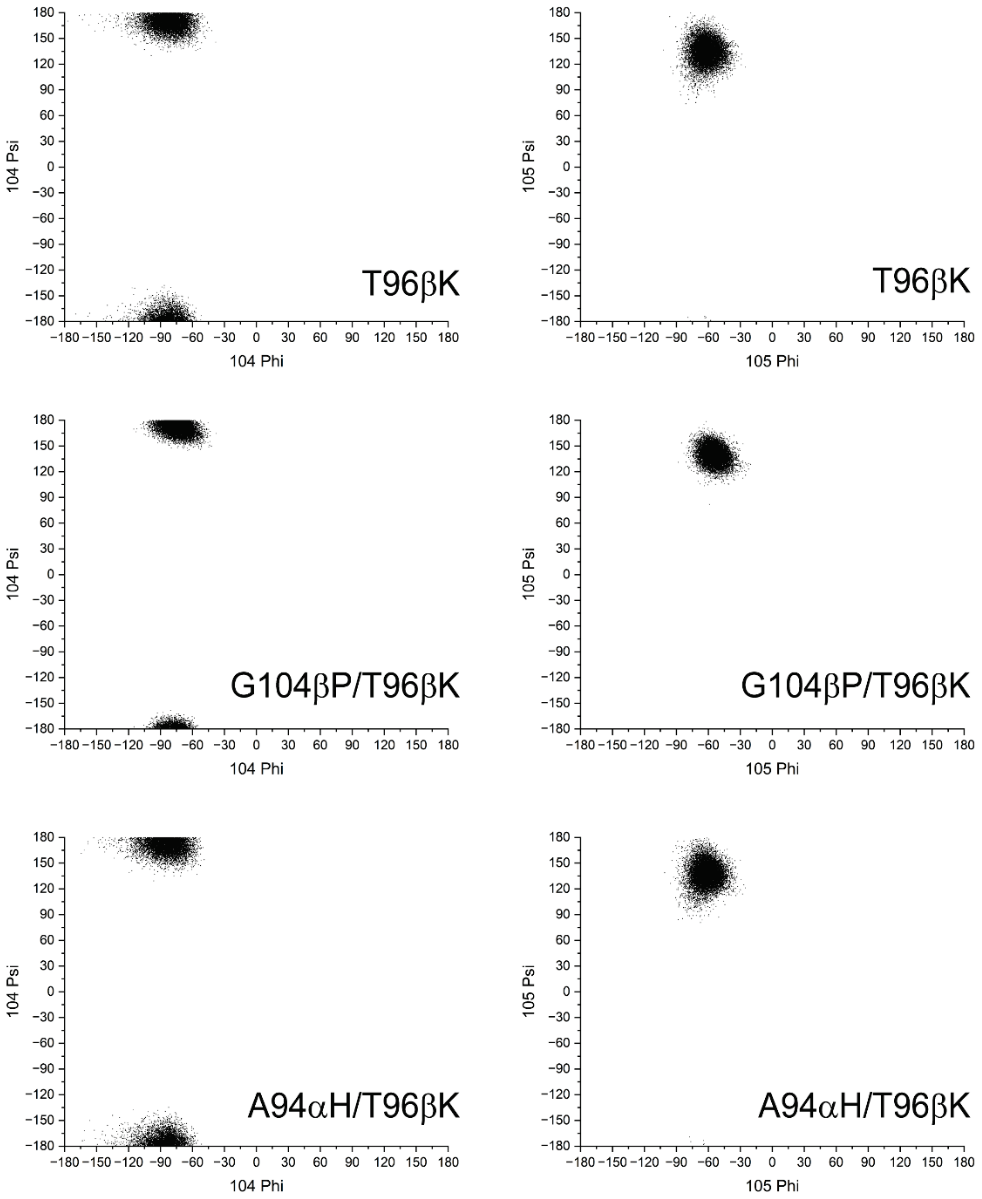

Supplementary Figure S3. ψ/ϕ and distributions for from the molecular dynamics simulations for positions 104β and 105β in the 868-Z11 T96βK (top), G104βP/T96βK (middle), and A94αH/T96βK (bottom) simulations.

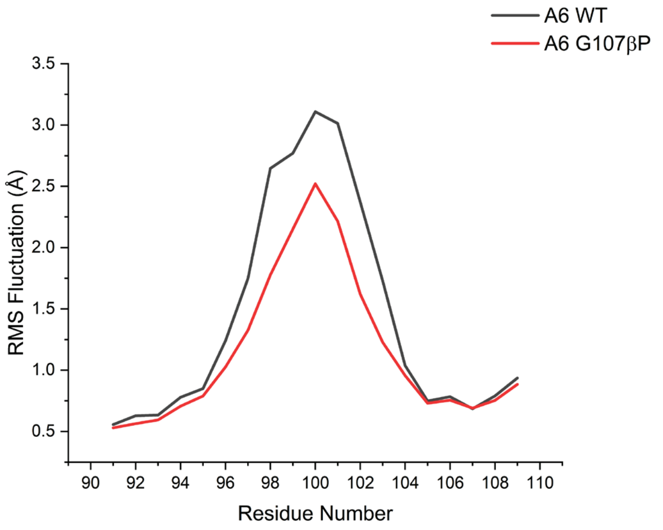

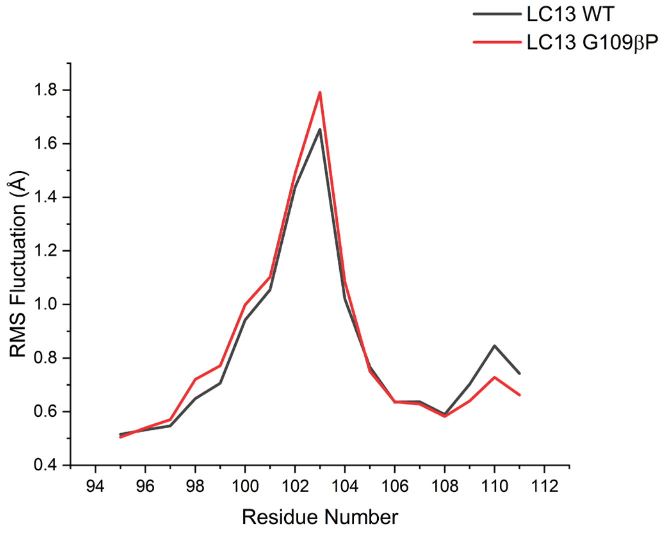

Supplementary Figure S4. RMS fluctuations for CDR3β of the A6 (top) and LC13 (bottom) TCRs. Eliminating the glycine-to-cysteine hydrogen by mutating Gly107β to Pro in A6 led to small reduction in the tip of the loop. The same mutation at Gly109β in LC13 was predicted to have much smaller consequences, with a small increase in fluctuations at the loop tip.
